# Household food security during the COVID-19 pandemic in urban and semi-urban areas in Indonesia

**DOI:** 10.1186/s41043-022-00285-y

**Published:** 2022-02-21

**Authors:** Ahmad Syafiq, Sandra Fikawati, Syilga Cahya Gemily

**Affiliations:** Center for Nutrition and Health Studies, Faculty of Public Health, University of Indonesia, Building F Level 2, Faculty of Public Health University of Indonesia, Depok, West Java Indonesia

**Keywords:** Food security, COVID-19 pandemic, Urban area, Semi-urban area

## Abstract

**Background:**

One of the impacts of the COVID-19 pandemic was the weakening of the community's economic condition. The weak economy of the community will have an impact on household food security. This study aims to determine food security in the COVID-19 pandemic situation and the impact of the pandemic on food security in urban and semi-urban areas.

**Methods:**

A cross-sectional study with a total sample of 517 people who live in urban (Jakarta) and semi-urban (Depok) areas. The research data was collected online and purposively through *Posyandu* cadres who have access to family/community. Food security was measured using HFIAS (Household Food Insecurity Access Scale) method, while impact of COVID-19 pandemic was categorized into two categories: impacted (reduced income and laid off) and less impacted (not reduced income and laid off). Data analysis used the Chi-square test and multiple logistic regression.

**Results:**

There were 65.0% of households with various level of food insecurity during the COVID-19 pandemic. The results of the multivariate analysis showed that family income during the COVID-19 pandemic (AOR = 4.2; CI = 2.7–6.7), the type of impact of the COVID-19 pandemic, i.e., reduced income and stopped working (AOR = 2.6; CI = 1.6–4.1), and the age of the respondent (AOR = 1.7; CI = 1.1–2.5) were significantly related to household food security during the pandemic after being controlled by husband's work status. Households with lower income had 4 times higher risk to experience food insecurity compared to those with higher income. Heavily impacted households (through reduced income and stopped working) had 3 times higher risk to experience food insecurity compared to those who did not. Additionally, we found that households with younger respondent (< 31 years old) had 2 times higher risk to experience food insecurity compared to those older counterparts.

**Conclusions:**

The COVID-19 pandemic impacted household food security in both urban and semi-urban areas through worsening employment status and income condition.

## Background

Currently, the world is facing a COVID-19 pandemic which come in waves, and it is difficult to predict when it will be ended. The COVID-19 pandemic has had a very broad impact, not only health and psychological impacts, but also impacts on the economic aspect of the society and the well-being of a nation. The implementation of measures to reduce infection such as physical distancing, work from home, travel ban, and other personal hygiene practices had affected and to a large extent, disrupted the economy [1]. Not a few companies finally terminated their employees to keep the company's financial health. The Ministry of Manpower of Republic of Indonesia reported that workforce affected by COVID-19 reached ± 3.05 million people as of early June 2020, and unemployment was estimated to reach 5.23 million [2]. Groups who were more at risk of losing their jobs and income are those from poor and vulnerable households (shop workers, waiters, cleaners, etc.) as well as those from the informal sector [3].

The weakened conditions of economics as a whole would certainly have impacts on purchasing power and consumption, especially in terms of food consumption. This will affect household food security. Studies had shown that low economic condition of family was significantly associated with household food insecurity [4–6]. The condition of low household food security will have an impact on child feeding. Studies also reported that food insecurity was significantly associated with child feeding practices [7–9]. Children living in food-secure households were significantly more likely to achieve appropriate feeding practices than children in food-insecure households [7].

Feeding the child should be in accordance with the nutrition recommendation because children simply need adequate nutrition to support growth and development. Nutrient intakes in toddlers, both macronutrient and micronutrient, has a major role in growth, and intake in sufficient quantities can prevent growth faltering in children [10–12]. If child feeding is not in accordance with the recommendation, it will affect the child's growth and cause nutritional problems such as stunting. Before the pandemic, the number of malnutrition in children under five in Indonesia was already high. Based on Basic Health Research (*Riskesdas*) data in 2018, the prevalence of stunting was 30.8%, underweight was 17.7%, wasting was 10.2% [13]. The high prevalence of malnutrition problems in Indonesia might be caused by many factors, one of them is the lack of energy and protein intake as a direct result of improper child feeding.

Pandemic was predicted to escalate malnutrition problems such as stunting, underweight and wasting among under-five children. In 2021, stunting was predicted to be increased to 32.5%, underweight to 23.0% and wasting to 10.5% [14]. One major cause of the increasing malnutrition rates due to COVID-19 pandemic was through reduced household income [15] which negatively affected buying power as well as reduced food availability and reduced household food security level [16]. Lower household food security directly affect food consumption and nutrient intakes of household members at varying degree.

This study aims to determine food security and the impact of the pandemic on food security in urban and semi-urban areas in Indonesia. The Jakarta area represents people from urban areas (urban) with middle to upper socio-economic level and the Depok area represents people in semi-urban areas with a more heterogeneous socio-economic, some were middle to upper and middle to lower.

## Methods

This study was quantitative research with a cross-sectional approach. The research was conducted in Jakarta and Depok in October–December 2020. Samples were taken from people who live in Jakarta and Depok in accordance with the inclusion criteria (have family that one of its members consisting of infant, toddlers, pregnant women, lactating mothers, and come from families with middle and low income (< minimum salary level Jakarta and Depok)). The selection of a family that has one of them is an infant/toddler/under-five/pregnant mother/lactating mother because the food security seen is from the vulnerable group in the first 1000 days whose nutrients must be met. The sample size in this study was obtained using the formula for the two-proportion difference hypothesis test sample, and based on previous research [17], it was found that the sample size was 500 people, and in this study, the respondents obtained were 517 people. The formula used was as follows [18]:

$$n = \frac{{\left\{ {z_{1 - \alpha /2} \sqrt {2P \left( {1 - P} \right)} + Z_{1 - \beta } \sqrt {P_{1} \left( {1 - P_{1} } \right) + P_{2} \left( {1 - P_{2} } \right)} } \right\}^{2} }}{{\left( {P_{1} - P_{2} } \right)^{2} }}$$

*Note* *n*: number of sample, $z_{1 - \alpha /2}$: *Z* score at 1–*α*/2 (90%) = 1.64, $Z_{1 - \beta }$: *Z* score at 80% power = 0.84, *P*_1_: Proportion of food security among low wife’s education = 0.556 [17], *P*_2_: Proportion of food security among high wife’s education = 0.445 [17], *P*: (*P*1 + *P*2)/2 = 0.501.

The dependent variable in this study was food security. Independent variables were the impact of the COVID-19 pandemic, wife's education, husband's education, wife's work status during the pandemic, husband's work status during the pandemic, number of family dependents during the pandemic, family income during the pandemic, family expenses during the pandemic, the number of people working during the pandemic and respondent's age. The impact of the COVID-19 pandemic in this study was categorized into two categories, namely affected (reduced income and stopped working) and less affected (other than reduced income and stopped working). Food security is measured using the HFIAS (Household Food Insecurity Access Scale) method, reflecting physical access (food availability at the household level) [19]. Respondents were given nine questions, each of which had a score of 0–3. In this method, food security was categorized into four levels, namely food security if the total score was 0–1, mild food insecurity if the total score was 2–7, moderate food insecurity if the total score was 8–14, and severe food insecurity if the total score was 15–27 [20]. From that categories, then categorized into 2, namely food security and food insecurity.

Data was collected purposively, namely respondents who live in the Jakarta and Depok areas and meet the research inclusion criteria were asked to fill out online questionnaires. Methods to contact respondent were through advertisement on social media and through list of mothers held by *Posyandu* cadres. Social media used to get contact with potential respondent were Facebook and Instagram. To anticipate fraud, where one respondent might fill out the questionnaire several times, screening was implemented and link to online questionnaire was personally distributed to candidate respondents. However, this step was not so effective because numbers of respondent who fill out the questionnaire were very small and data collection was very slow. Therefore, an additional method of contacting respondents was done through gathering list of mothers from Posyandu cadres. *Posyandu* cadres from Jagakarsa Village, Tanjung Barat Subdistrict in Jakarta and Beji, Beji Subdistrict in Depok were selected purposively based on their willingness to collaborate in conducting research in the pandemic era and their willingness to share link to screening questionnaire to families in their environment. Furthermore, respondents (from the list of *Posyandu* cadres) who had filled out the screening link were checked and if they met the inclusion criteria, a complete questionnaire link was given. This second method gave better results in term of participation and representativeness.

After the data were cleaned (including treatment for missing data, in which there was none), univariate, bivariate, and multivariate analyses were conducted. Chi-square test was used in bivariate analysis. Variables with *p* value less than 0.25 in bivariate analysis were included in the multivariate analysis using multiple logistic regression. Odds ratios with 95% confidence interval were calculated to determine the association between food security and other independent variables. Collinearity and interaction were examined, and no significant collinears and interaction were found. Test results were significant when *p* value is ≤ 0.05.

## Results

A total of 517 respondents participated in this study. Respondents obtained from advertisement on social media were 167 people (115 in Jakarta and 52 in Depok). Respondents obtained from the list of *Posyandu* cadres were 350 people (143 in Jakarta and 207 in Depok (Table 1).

**Table 1:** Number of respondents by data source and region

Data source	Region		Total
	Jakarta	Depok	
Social media	115 (44.6%)	52 (20.1%)	167 (32.3%)
*Posyandu* cadres	143 (55.4%)	207 (79.9%)	350 (67.7%)
Total	258 (100%)	259 (100%)	517 (100%)

As many as 75.2% of households in study areas experienced the impact of the COVID-19 pandemic. The type of impact occurred in both urban and semi-urban areas were relatively similar. Impact mostly felt was the reduction in income (felt by 63.2% respondent in Jakarta and 62.2% in Depok). Other impacts including not being able to leave the house (18.2% in Jakarta and 17.8% in Depok), stopped work (13.6% in Jakarta and 11.6% in Depok), increasing price of household necessities (4.7% in Jakarta and 6.2% in Depok), increasing food prices (felt only in Depok, 2.3%), and lack of public transportation (felt only in Jakarta, 0.4%).

The impact of pandemic started to be felt in both Jakarta and Depok in March 2020 (62.8% and 68.3%, respectively). In terms of food shortages during the pandemic, half of the respondent said they sometimes experienced it (50.8% in Jakarta and 47.1% in Depok). The cause of food shortages was mainly due to reduced family income (81.3% in Jakarta and 81.6% in Depok). The results showed that in total, 15.5% of households were categorized as having severe food insecurity, 28.2% having moderate food insecurity, and 21.3% having mild food insecurity, while 35.0% of respondents could be categorized as having adequate food security (Table 2).

**Table 2:** Situation of the impact of the pandemic and food security during the pandemic

Variable	Jakarta	Depok	Total
*Impact of the COVID-19 pandemic*			
Affected	198 (76.7%)	191 (73.7%)	389 (75.2%)
Less affected	60 (23.3%)	68 (26.3%)	128 (24.8%)
*First impact suffered*			
Income reduced	163 (63.2%)	161 (62.2%)	324 (62.7%)
Can't leave the house	47 (18.2%)	46 (17.8%)	93 (18.0%)
Stopped working	35 (13.6%)	30 (11.6%)	65 (12.6%)
Expensive price	12 (4.7%)	16 (6.2%)	28 (5.4%)
Expensive food prices	0 (0.0%)	6 (2.3%)	6 (1.2%)
Others (lack of public transportation)	1 (0.4%)	0 (0.0%)	1 (0.2%)
*First impact of the pandemic*			
March 2020	162 (62.8%)	177 (68.3%)	339 (65.6%)
April 2020	60 (23.3%)	53 (20.5%)	113 (21.9%)
May 2020	1 (0.4%)	0 (0.0%)	1 (0.2%)
June 2020	8 (3.1%)	3 (1.2%)	11 (2.1%)
July 2020	4 (1.6%)	3 (1.2%)	7 (1.4%)
August 2020	3 (1.2%)	1 (0.4%)	4 (0.8%)
September 2020	1 (0.4%)	0 (0.0%)	1 (0.2%)
October 2020	19 (7.4%)	22 (8.5%)	41 (7.9%)
*Food shortages during pandemic*			
Yes always	28 (10.9%)	21 (8.1%)	49 (9.5%)
Yes, often	28 (10.9%)	31 (12.0%)	59 (11.4%)
Yes, sometimes	131 (50.8%)	122 (47.1%)	253 (48.9%)
No	71 (27.5%)	85 (32.8%)	156 (30.2%)
*Cause of food shortage*			
Expensive food price	18 (9.6%)	18 (10.3%)	36 (10.0%)
Decreased income	152 (81.3%)	142 (81.6%)	294 (81.4%)
Don't have income	16 (8.65)	14 (8.0%)	30 (8.3%)
*Food security during a pandemic*			
Severe food insecurity	45 (17.4%)	35 (13.5%)	80 (15.5%)
Moderate food insecurity	77 (29.8%)	69 (26.6%)	146 (28.2%)
Mild food insecurity	54 (20.9%)	56 (21.6%)	110 (21.3%)
Adequate food security	82 (31.8%)	99 (38.2%)	181 (35.0%)
*Food security during a pandemic*			
Food insecurity at various level	176 (68.2%)	160 (61.8%)	336 (65.0%)
Adequate food security	82 (31.8%)	99 (38.2%)	181 (35.0%)

The data showed that during pandemic in Jakarta and Depok, almost all families (90%) reduced the variety of food consumption when they experiencing food shortages. In addition, at the family level, an average of 83.9% of respondents also had a condition of not eating their preferred food, 72.0% consuming less food than needed, 68.4% consuming less food in a day, 65.1% consuming food that they do not want to eat, 28.8% did not eat anything in a day, 23.0% had experienced sleep in hunger at night and 12.5% did not eat anything a day and night because there was not enough food (Table 3).

**Table 3:** Food consumption conditions in households experiencing food shortage during a pandemic

Variable	Jakarta	Depok	Total
*Not eating favorite foods due to lack of resources*			
Yes	157 (84.0%)	146 (83.9%)	303 (83.9%)
No	30 (16.0%)	28 (16.1%)	58 (16.1%)
*Eating less varied food due to lack of resources*			
Yes	168 (89.8%)	325 (90.2%)	325 (90.0%)
No	19 (10.2%)	17 (9.8%)	36 (10.0%)
*Eating food that you don't want to eat due to lack of resources to get other food*			
Yes	126 (67.4%)	109 (62.6%)	235 (65.1%)
No	61 (32.6%)	65 (37.4%)	126 (34.9%)
*Eating less food than needed because there is not enough food*			
Yes	133 (71.1%)	127 (73.0%)	260 (72.0%)
No	54 (28.9%)	47 (27.0%)	101 (28.0%)
*Eating less food in a day because there is not enough food*			
Yes	129 (69.0%)	118 (67.8%)	247 (68.4%)
No	58 (31.0%)	56 (32.2%)	114 (31.6%)
*Not consuming anything as a result of food unavailability at home due to lack of resources to obtain food*			
Yes	58 (31.0%)	46 (26.4%)	104 (28.8%)
No	129 (69.0%)	128 (73.6%)	257 (71.2%)
*Sleeping in hungry condition at night because there is not enough food*			
Yes	46 (24.6%)	37 (21.3%)	83 (23.0%)
No	141 (75.4%)	137 (78.7%)	278 (77.0%)
*Do not eat anything day and night because there is not enough food*			
Yes	28 (15.0%)	17 (9.8%)	45 (12.5%)
No	159 (85.0%)	157 (90.2%)	316 (87.5%)

Changes in food consumption during the pandemic also occurred in vulnerable groups such as infants, toddlers, under-five, pregnant women, and lactating mothers in the family. Based on research data, it was known that changes in eating patterns were more common in pregnant women, which was 68.5%. Most of the changes in eating that occurred were in the reduction in the types of food eaten (Table 4), and one important finding was 46.7% mothers reduced consumption of fruits (Table 5).

**Table 4:** Changes in the diet of vulnerable groups in households during the pandemic

Variable	Infant	Toddler	Under-five	Pregnant women	Lactating mother
*Changes in family diet*					
Yes	18 (16.2%)	51 (44.0%)	138 (43.0%)	50 (68.5%)	87 (46.3%)
No	93 (83.8%)	65 (56.0%)	183 (57.0%)	23 (31.5%)	101 (53.7%)
*Changes in eating patterns that occur*					
The type of food eaten is reduced	9 (50.0%)	35 (68.8%)	88 (63.8%)	30 (60.0%)	54 (62.1%)
Reduced amount of food eaten	7 (38.9%)	10 (19.6%)	30 (21.7%)	9 (18.0%)	23 (26.4%)
Decreased number of meals per day	1 (5.6%)	4 (7.8%)	18 (13.0%)	11 (22.0%)	9 (10.3%)
Others (unable to buy nutritious food, eat potluck, change brands)	1 (5.6%)	2 (3.9%)	2 (1.4%)	0 (0.0%)	1 (1.1%)

**Table 5:** Food groups with reduced consumption reported by pregnant women

Food groups	Frequency (percentage)
Staple food	3 (10.0%)
Protein source food	7 (23.3%)
Fruits	14 (46.7%)
Dairy	6 (20.0%)

Multivariate analysis was carried out by including nine independent variables, namely the impact of the COVID-19 pandemic, wife's education, husband's education, wife's work status during the pandemic, husband's work status during the pandemic, number of family dependents during the pandemic, family income during the pandemic, family expenses during the pandemic and respondent's age. The results of the analysis showed that variables significantly related to food security were family income during the COVID-19 pandemic (AOR = 4.2; CI = 2.7–6.7), the type of impact of the COVID-19 pandemic (AOR = 2.6; CI = 1.6–4.1), and respondent's age (AOR = 1.7; CI = 1.1–2.5) after being controlled by husband's work status during the pandemic (Table 6). Households with lower income have 4 times higher risk to experience food insecurity compared to those with higher income. Heavily impacted households (through reduced income and laid off) have 3 times higher risk to experience food insecurity compared to those who did not. Additionally, we found that households with younger respondent (< 31 years old) have 2 times higher risk to experience food insecurity compared to those older respondents.

**Table 6:** Multivariate final model

Variable	*P* value	OR (95% CI)
Impact of the COVID-19 pandemic	< 0.001	2.6 (1.6–4.1)
Family income during the pandemic	< 0.001	4.2 (2.7–6.7)
Respondent age	0.011	1.7 (1.1–2.5)
Husband's work status	0.171	1.8 (0.8–4.1)

## Discussion

Results of this study indicate that there was no significant difference in the impact of the pandemic on the community's economy between urban and semi-urban areas. In both urban and semi-urban areas, the impact of the COVID-19 pandemic most felt (felt by 62.7% respondents) was reduction in income. Similar thing was also reported by a study conducted in Vietnam which stated that the majority of respondents (66.9%) had a decrease in household income due to COVID-19 [15]. A study on American society conducted by the Pew Research Center [21] also reports that 60% of respondents had lower incomes than before the COVID-19 pandemic due to job losses, reduced worked hours, or pay cuts.

Regarding food security, this study showed that only 35% of respondents had adequate food security, the remaining 65.0% of respondents had various level of food insecurity during the COVID-19 pandemic. Food insecurity during the COVID-19 pandemic also occurred in various countries in the world with the relatively similar prevalence. In Bangladesh, there was an increase in the number of families experiencing food insecurity during the COVID-19 pandemic lockdown by 51.7% [22]. Adams et al. [23] in California also reported that there was an increase in food insecurity conditions during the COVID-19 pandemic. A study conducted in the USA showed that out of 3219 respondents, 32.3% of households had very low food security and 67.7% of households had low food security since COVID-19 [24].

Food shortages during the pandemic affected household food consumption. In this study, households that had food shortages tend to consume less varied foods. In addition, there were those who eat less food and some even do not eat any food in a day. Changes in household food consumption during the pandemic also occurred in Uganda and Kenya. Research conducted by Kansiime [16] indicated that during a pandemic COVID-19, a reduction in the frequency of consumption of a variety of food groups and mainly occurs in low-income families. Jafri et al. [25] also reported that during the COVID-19 pandemic there was less access to food so respondents tended to reduce the size and amount of food.

Changes in food consumption among vulnerable groups such as infants, toddlers, under-five, pregnant women, and lactating mothers also occurred in this study. It is of important note that pregnant women were the respondent who mostly experienced changes in food consumption during the pandemic in this study, they mostly reduced variety of food eaten. This reduction in variety of food among pregnant women was worrying because it will affect the adequacy of nutrient intakes such as protein, fat, iron, calcium, and other micronutrients that could affect the growth and development of fetus [26]. In this study, reduction in fruit consumption was the most common. This might reduce the intakes of folic acid, vitamin C and magnesium, all are micronutrients deemed as important for pregnant mothers [27]. Vitamin C would also influence absorption of iron and thus very important to overcome anemia, problem commonly suffered by pregnant women [26, 28]. Studies showed that fruit consumption also affect infant’s birth weight and increasing fruit consumption would reduce low birth weight risk [27, 29, 30].

Research conducted by Jafri et al. [25] reported that vulnerable groups such as children, pregnant women and the elderly were struggling to obtain adequate food during the COVID-19 pandemic, and one way to cope with this was by reducing the amount of food eaten. Changes in food consumption in vulnerable groups during the pandemic in this study pose high risk of the escalation of malnutrition problems, such as stunting [31]. On the other hand, parents were also at risk of experiencing malnutrition and decreased immunity which would in turn, increase the morbidity and mortality, especially among older parents [32].

This study reveals that family income during pandemic had a significant relationship with household food security. Households with low income (below the Jakarta/Depok minimum salary level) were 4 times more likely to experience food insecurity than households with higher income. Study in Bangladesh show that income loss was positively correlated with household food insecurity [33]. Family income directly affected family's purchasing power [34] and low family income tended to lead a less varied food [35, 36].

The type of impact of pandemic, in this case reduced income and stopped working, also had a significant relationship with food security. This finding is in line with research conducted by Kundu et al. [37] which showed that employment and income are potential predictors of low food security scores. Unicef [38] also emphasized that job losses and reduced income hindered access to food.

Another variable that had significant relationship with household food security was the age of the respondents. In this study, respondents aged < 31 years had a 2 times higher risk of experiencing food insecurity compared to their older counterpart. Similar results were also reported by Elsahoryi et al. [39] in Jordan, age was associated with food insecurity status, with younger respondents (18–30 years old) were 1.8 times more likely to experience severe food insecurity. Research by Abdullah et al. [40] in Malaysia, however, showed different result, where households with older household's heads were more food insecure than households with younger household's heads. Age is associated with the economic condition of the family, namely the age of older respondents tends to have a more established economy when compared to younger ages [40].

Husband's work status during the pandemic in this study became the control variable in the relationship between the independent variables and food security. The husband's work affected the amount of income received by the family which then affected the household's food security. Charvadeh et al. [41] reported that during the COVID-19 outbreak, the household head's work status was directly related to food security. Research in Bangladesh also showed that husband's employment status was significantly related to food security [37].

Results of this study could be applicable to other areas with similar setting, i.e., urban or semi-urban with typical socio-economic status of developing countries. It might be different, though, with rural areas where food network among families were more dense and stronger. There are some limitations of this study. First, this study was conducted online with its inherent flaw regarding possibility of distorted response which might be different to face-to-face interview. However, we expect the bias was non-differential due to similar environmental and socio-biographic characteristics of the respondents. Second, details of food consumption could not be captured perfectly since we could not use conventional food consumption instruments such as Food Frequency Questionnaire (FFQ) considering the burden of online survey.

## Conclusion

The COVID-19 pandemic had an impact on decreasing the level of household food security through negative impacts on people's work and income in both urban and semi-urban areas. In this study, the variables that were significantly related to food security were family income during the COVID-19 pandemic (AOR = 4.2; 95%CI = 2.7–6.7), the type of impact of the COVID-19 pandemic, i.e., reduced income and stopped working (AOR = 2.6; 95%CI = 1.6–4.1), and respondent's age (AOR = 1.7; 95%CI = 1.1–2.5) after being controlled by husband's work status during the pandemic.

## Recommendation

It is suggested to conduct more research by adding variables related to nutritional status especially for those vulnerables such as under-five children and pregnant women. There are challenges to develop a culturally sensitive instrument to measure food consumption via online survey. Government should provide more food security related content in information, education and communication on pandemic targeted to the communities. Especially related to coping strategies as not to sacrifice nutritional needs of vulnerable groups, e.g., pregnant women. Moreover, special attention should be paid to assist social aids including food aids to consider vulnerable groups in the family.

## Data Availability

The datasets during and/or analyzed during the current study available from the corresponding author on reasonable request.
